# Sorghum–Insect Composites for Healthier Cookies: Nutritional, Functional, and Technological Evaluation

**DOI:** 10.3390/foods9101427

**Published:** 2020-10-09

**Authors:** Temitope D. Awobusuyi, Muthulisi Siwela, Kirthee Pillay

**Affiliations:** Department of Dietetics and Human Nutrition, University of KwaZulu-Natal, Pietermaritzburg 3209, South Africa; temmybusuyi@gmail.com (T.D.A.); pillayk@ukzn.ac.za (K.P.)

**Keywords:** protein energy malnutrition, insect, sorghum, wheat

## Abstract

Protein-energy malnutrition (PEM) is a major health concern in sub-Saharan Africa (SSA). Relying on unexploited and regionally available rich sources of proteins such as insects and sorghum might contribute towards addressing PEM among at-risk populations. Insects are high in nutrients, especially protein, and are abundant in SSA. Sorghum is adapted to the tropical areas of SSA and as such it is an appropriate source of energy compared with temperate cereals like wheat. It is necessary to assess whether cookies fortified with sorghum and termite would be suitable for use in addressing PEM in SSA. Whole grain sorghum meal and termite meal were mixed at a 3:1 ratio (*w*/*w* sorghum:termite) to form a sorghum–termite meal blend. Composite cookies were prepared where the sorghum–termite blend partially substituted wheat flour at 20%, 40%, and 60% (sorghum–termite blend:wheat flour (*w*/*w*). The functional and nutritional qualities of the cookies were assessed. Compared with the control (100% wheat flour), the cookies fortified with sorghum and termite had about double the quantity of protein, minerals, and amino acids. However, with increased substitution level of the sorghum–termite blend, the spread factor of the cookies decreased. There is a potential to incorporate sorghum and termite in cookies for increased intake of several nutrients by communities that are vulnerable to nutrient deficiencies, especially PEM.

## 1. Introduction

The World Health Organization (WHO) estimates that about 60% of all deaths occurring among children under five years of age in developing countries could be attributed to malnutrition [1]. Protein-energy malnutrition (PEM) results from deficiency in dietary protein and/or energy in varying proportions [2]. Sub-Saharan Africa (SSA) continues to lead in bearing the brunt of PEM and globally, the prevalence has continued to rise up to 47.3% with the worst increase occurring in regions of Africa [3,4]. Therefore, an improvement in nutrition is needed to decrease the high mortality and morbidity rates associated with PEM [5].

Sorghum, a drought-tolerant staple, contributes to the diet of over half a billion people in the regions, where maize struggles to grow if there are very limited agronomic intervention technologies [6,7]. Baked products, such as bread and cookies are part of the leading foods world-wide, including in the sub-Saharan African region. Therefore, they are the most appropriate vehicles to deliver vital nutrients, for example protein to vulnerable populations [8]. In SSA, the limitation of producing wheat due to the less conducive climatic conditions and the exorbitant demand on forex for its importation creates a necessity to try to substitute wheat with the well adapted cereals like sorghum in baked products [9]. However, sorghum grain is low in protein content, while the essential amino acids lysine, threonine, and tryptophan are limited [10,11]. Hence, if sorghum were to be used to partially substitute wheat in baked goods, it would be necessary to find an accessible, yet high quality source of nutrients, including protein, to complement it.

Insects are a traditional source of food in several parts of the world. They are especially rich in protein, calcium, iron, and zinc [12,13]. The energy content of insects is comparable to that of meat, except for pork, because of its particularly high fat content [14]. Furthermore, given that food insecurity is prevalent in SSA, the use of insects in this region, where they are already being consumed, although not at a nutritionally significant level, should be promoted to serve as an alternative protein source, in particular. Thus, the incorporation of insect in popular, staple foods to complement staple cereal grains should be considered.

Cookies are an energy dense and shelf-stable, popular baked ready-to-eat snack, consumed by both children and adults globally [15,16]. The main ingredients in cookie baking include wheat flour, fat, sugar, butter, and water. Other added ingredients may be optional or added to improve organoleptic attributes [17]. With the afore-mentioned nutritional advantages of insects and the popularity of cookies, it might be advantageous to complement sorghum with insects in partial replacement of wheat flour in cookies to contribute to addressing PEM in developing regions, especially SSA.

Sorghum–legume cookies in which sorghum was combined with sunflower and peanut flours, to increase the protein content of the cookies have been reported [18]. In addition, Mridula et al. [19] reported that acceptable cookies could be developed with wheat–sorghum composite flours with up to 50% sorghum substitution level. Several studies have demonstrated the potential for supplementing wheat flour with sorghum in bread, and cookies, and other snacks [9,20,21]. The influence of finger millet flour [22], fibers from different cereals [23], maltodextrin, and guar gum [24] on the rheological properties of dough and quality of cookies has also been reported. However, it appears that the compositing of wheat, sorghum, and termite to make cookies has not been reported. Therefore, this study aimed to determine the effect of partially substituting wheat flour with a sorghum–termite blend on the nutritional composition and functional properties of cookies.

## 2. Materials and Methods

### 2.1. Preparation of Sorghum and Termite Meal Blend

Winged termites (*Macrotermes belliscosus*), harvested during the harmattan season were purchased from Oja-oba main market in Ondo State, Nigeria, and used in this study. The termites were de-winged and cleaned three times to remove soil and dirt. They were oven-dried at 40 °C for 8 h [25]. Dried insects were milled into a meal with a blender to a particle size of ≤1 mm, vacuum packed, labelled and stored at −4 °C until analysis. The termites were washed, dried in the oven, and milled into a meal. Sorghum grain was purchased and cleaned to make sure they were free of dirt. A mill fitted with a 0.4 mm screen was used to grind whole grain sorghum meal into a meal [9]. Both the sorghum and termite meal were used to substitute wheat flour at varying proportions (Table 1).

### 2.2. Methods

#### 2.2.1. Preparation of Cookie Samples

Cookies fortified with sorghum and termite as well as the control were prepared according to the method described by de Jager [26], with minimal modification. The sorghum meal and insect meal were mixed at a 3:1 (*w*/*w*) ratio to form a sorghum–termite blend. The ratio 3:1 was chosen after preliminary trials in the laboratory revealed that other substitution levels of sorghum and termite resulted in cookies that were too brittle. Experimental cookies were prepared where wheat flour was partially substituted with different proportions of the sorghum–termite blend, 20%, 40%, and 60% (*w*/*w*), separately. Cookies (100% wheat) in which no sorghum or insect was added served as the control (Table 1). About 200 g of sugar, 5 mL of vanilla essence, 5 mL of salt, 50 g of powdered milk, and 10 mL baking powder were sieved and mixed together with 480 g wheat flour for three to five minutes. About 250 g of margarine was added to the mixture and kneaded for two minutes to form a firm dough. The dough was rolled out, cut into desired shapes, and transferred into the oven. Cookies were baked at 150° C in a preheated oven for 20 min. Cookies were crushed to a particle size of ≤1 mm for chemical analyses and then stored.

#### 2.2.2. Nutritional Composition

The nutritional composition of cookies was determined by standard methods stated below.

#### Ash

Ash was determined using the Association of Official Analytical Chemists (AOAC) official method 923.03 [27].

#### Protein

Crude protein was measured using the AOAC official method 968.06 [28].

#### Glycemic (Available) Carbohydrate Content

Glycemic carbohydrates were calculated by difference.

#### Fat

Fat content was determined according to the AOAC Official Method 920.39C [29].

#### Fiber

Fiber was done based on the method reported by Saha et al. [22].

#### Gross Energy

Gross energy was determined according to the AOAC Official Method 935.42 [30].

#### Selected Minerals

Mineral content was determined by the AOAC Official Method 6.1.2 [31].

#### Amino Acids

The amino acid profile of the cookie samples was analysed by the Waters API Quattro Micro Method, which consists of a column C18, 1.7 μm, 2.1 × 100 mm and a binary solvent manager. Samples (400 mg) were subjected to AccQ-Tag Ultra Derivatization kit (Waters^TM^, Johannesburg, South Africa); 10 μL of the undiluted sample was added to the Waters AccQ-Tag kit constituents and placed in a heating block at a temperature of 55 °C for 10 min. Injection volume was 1 μL and gradient separation was performed using Solvents A and B from the Waters Accutag kit. 

#### In Vitro Protein Digestibility

In vitro protein digestibility was determined using the method described by Hamaker et al. [32].

#### 2.2.3. Physical Characteristics

Physical quality parameters of the cookie samples, such as diameter, thickness, spread ratio, and spread factor were determined using the procedure of the American Association of Cereal Chemists [33].

#### Texture

**Figure 1:**
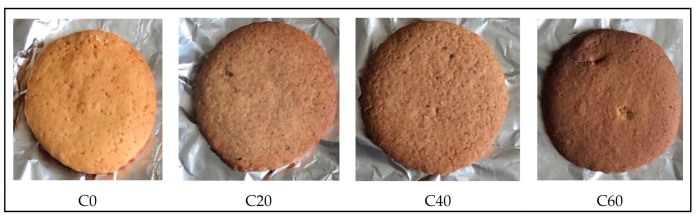
Cookies fortified with sorghum and termite. C0: control (100% wheat cookies); C20: 15% sorghum and 5% termite substitution level; C40: 30% sorghum and 10% termite substitution level; C60: 45% sorghum and 15% termite substitution level.

Texture analysis of cookies fortified with sorghum and termite (Figure 1) was done using a TA-XT plus 100C model texture analyzer (Stable Micro Systems, Godalming, UK). The cookies were measured for hardness and fracturability using a three-point bend rig attachment at a 3.0 mm/s cross head speed for a 5 mm distance and a 5 kg load cell.

#### 2.2.4. Functional PropertiesWater and Oil Absorption Capacity

##### Water and Oil Absorption Capacity

The water and oil absorption capacities were determined by the method of Sosulski et al. [34].

##### Bulk density

The process reported by Okaka and Potter [35] was used to determine the bulk density of the cookie flours.

#### 2.2.5. Statistical Analysis

The resulting data was analysed using the Statistical Package for Social Science (SPSS version 20.0 SPSS Inc., Chicago, IL, USA). One-way analysis of variance (ANOVA) was done; and separation of means was by Fisher Least Significance Difference (LSD) test. A *p*-value of ≤0.05 was considered significant.

## 3. Results

### 3.1. Proximate Composition

Protein, fat, and carbohydrates constitute the major nutrients in cookie samples (Figure 1). The proximate composition of the cookies fortified with the sorghum–termite blend is presented in Table 2. As expected, the highest protein content was observed in cookies containing 60% sorghum and termite substitution level, which contained the most insect. The results showed that fat content increased with increasing concentrations of the sorghum–termite blend. The fiber and ash content of cookie samples fortified with the sorghum–termite blend was also significantly higher than the control. Carbohydrate content was higher in the control cookies than in the cookies fortified with the sorghum–termite blend. The gross energy of the cookies fortified with the sorghum–termite blend was higher than the control.

### 3.2. Mineral Composition

The minerals abundant in the cookie samples were iron, phosphorus, and magnesium (Table 3). The incorporation of the sorghum–termite blend substantially increased the mineral concentration of the cookies. Zinc and iron were abundant in the cookie samples fortified with the sorghum–termite blend. Cookies containing 60% of the sorghum–termite blend had the highest concentration of these minerals compared with the cookie samples with lower concentrations of the sorghum–termite blend.

### 3.3. Amino Acid Content

Table 4 shows that cookies containing the sorghum–termite blend had substantial amounts of amino acids and lysine and leucine were the major amino acids present. Cookies fortified with the sorghum–termite blend showed the highest increase across all amino acids when compared with the control.

### 3.4. In Vitro Protein Digestibility

The in vitro protein digestibility (IVPD) results are presented in Table 5. The addition of the sorghum–termite blend improved the digestibility of cookies, which increased by 23.8% with the incorporation of the sorghum–termite blend from 67% to 83%.

### 3.5. Physical Characteristics

The physical characteristics of cookies fortified with the sorghum–termite blend are shown in Table 6. The results showed that the addition of sorghum–termite blend reduced the weight of cookies, compared with the control. Cookies fortified with 60% sorghum–termite blend (24.5 g) recorded the highest weight loss when compared with the control (29.5 g). There was a progressive increase in the thickness of the cookies fortified with the sorghum–termite blend (7.5 mm, 7.7 mm, and 7.9 mm for sample C20, C40, and C60, respectively), when compared with the control (7.3 mm).

### 3.6. Texture and Colour

The effect of incorporating sorghum and termite on the texture and colour of cookies is shown in Table 7. The results showed that as the substitution level of the sorghum–termite blend increased, the cookie hardness decreased. Further, cookies became darker with increasing concentration of the sorghum–termite blend. The L* (lightness) values decreased and the a* (redness) values increased, while b* (yellowness) values were similar across all cookie samples.

### 3.7. Water and Oil Absorption Properties of Cookie Flours

**Figure 2:**
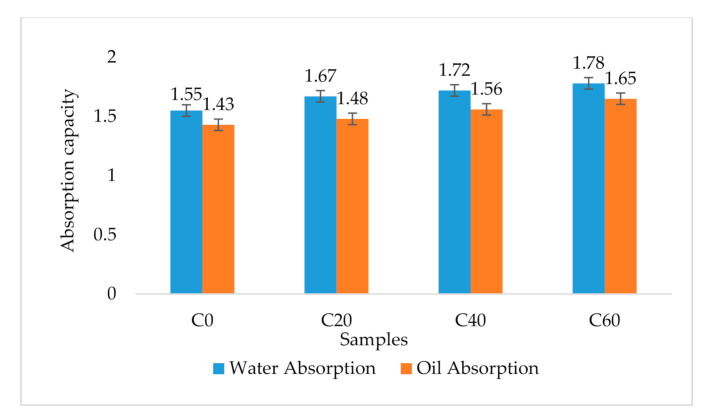
Water and oil absorption capacity of cookie flours. Water absorption is expressed as g water/g flour. Oil absorption is expressed as g oil/g flour. Error bar values are actual values obtained. C0: control (100% wheat cookies); C20: 15% sorghum and 5% termite substitution level; C40: 30% sorghum and 10% termite substitution level; C60: 45% sorghum and 15% termite substitution level.

The oil absorption capacity (OAC) varied between 1.43 and 1.65 g oil/g flour (Figure 2) while the water absorption (WAC) varied from 1.55 g to 1.78 g water/g flour. The results revealed that the higher the sorghum and termite substitution level, the higher the absorption capacities of the flours.

### 3.8. Bulk Density of Cookie Flours

**Figure 3:**
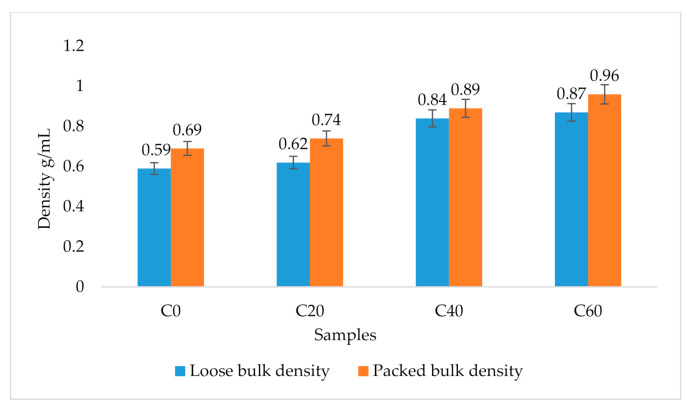
Bulk density of cookie flours. Error bar values are actual values obtained. C0: control (100% wheat cookies); C20: 15% sorghum and 5% termite substitution level; C40: 30% sorghum and 10% termite substitution level; C60: 45% sorghum and 15% termite substitution level.

The packed bulk density (PBD) and loose bulk density (LBD) varied (Figure 3). The PBD was 0.69 g/mL for the control and 0.96 g/mL for the 60% composite flour containing the sorghum–termite blend, which was much higher than the control. The LBD ranged between 0.59 and 0.87 g/mL. All composite flours containing wheat, sorghum and termite had relatively higher LBD than the wheat flour (control).

## 4. Discussion

### 4.1. Proximate Composition

Protein content was significantly higher in all cookies fortified with the sorghum–termite blend (36.4 to 41.0 g/100 g), compared with the control (10.5 g/100 g). This result agrees with Koffi-Niaba et al. [36], who reported that supplementing sorghum with termite flour significantly improved the protein content from 9.6% in the control to 21.7%. A report by Kinyuru et al. [37] working on the nutritional quality of wheat buns enriched with edible termites also found that the buns showed a significant increase in protein content (47.5%), when compared with buns without termite substitution. It has been reported that fortifying sorghum with defatted soy flour significantly enhanced the protein quality and content of cookies [21]. Similar results have also been reported by Omoba and Omogbemile [38]. Further, as the concentration of the sorghum–termite blend increases, a substantial increase was observed in the fat content. This is expected as termites, the insect used in this study ranked among the highest in fat concentration [39,40]. Fibre also recorded a significant increase across all cookies containing sorghum–termite blend due to the addition of insects (10.2 to 13.5 g/100 g). Fiber is beneficial in the human diet to reduce the risk of heart disease, blood pressure, obesity, and lower cholesterol levels [41].

The ash content of the cookie samples fortified with the sorghum–termite blend (3.5 to 4.2 g/100 g) was significantly higher than the control (1.7 g/100 g). However, C0 (control), had the highest carbohydrate content (54%). Cookies containing 60% sorghum–termite blend had the highest energy value (2217.6 kJ/100 g) and the lowest was recorded in the control (C0) (gross energy: 1180.2 kJ/100 g). This result could be attributed to the high fat content of the experimental cookies. The energy value of edible insects has been reported to depend mainly on their fat content [38]. Similar results were reported by Koffi-Niaba et al. [36], they obtained between 1626 kJ/100 g and 1712 kJ/100 g in sorghum-based cookies fortified with termites. Overall, these are promising results and developing countries, where PEM remains a major problem, could benefit from the consumption of these cookies.

### 4.2. Mineral Composition

Cookie samples fortified with the sorghum–termite blend had increased mineral content compared with the control (Table 3). The calcium content was significantly higher (5.5 to 10.8 mg/100 g) when compared with the control (2.5 mg/100 g). The iron content varied significantly, with the highest level (37.4 mg/100 g) found in sample C60 (45% sorghum, 15% insect substitution level). Although insects are known to have high levels of minerals [40], the higher levels noted in this study could also be partially attributed to the high iron content in sorghum [42]. Zinc levels were also significantly high across all cookies fortified with the sorghum–termite blend (8.4 to 14.8 mg/100 g), compared with the control (2.5 mg/100 g). This supports Yhoungaree et al. [43], who stated that insects are a valuable source of iron and zinc. A previous study used caterpillar cereal to prevent anemia and stunting in infants. The authors found that the caterpillar cereal produced had appropriate macro and micronutrient contents and concluded that it could be used for complementary feeding [44]. This further supports the results in this study, which showed that consumption of insects, could prove to be a valuable measure to combat deficiencies of iron and zinc in developing countries.

In this study, phosphorous content was highest in the cookie sample containing 60% sorghum–termite blend (37.6 mg/100 g) and lowest in the control (0.8 mg/100 g). Magnesium ranged from 24.3 to 33.5 mg/100 g in cookies fortified with the sorghum–termite blend, which was significantly higher than the control (1.8 mg/100 g). Copper ranged from 0.8 mg/100 g in the control to 3.8 mg/100 g in sample C60 (45% sorghum and 15% termite substitution level). Manganese ranged between 13.2 and 24.5 mg/100 g in cookies fortified with the sorghum–termite blend, which was higher than the control (1.2 mg/100 g). Overall, the addition of sorghum and termite substantially increased the mineral profile of the cookies. This study demonstrates the potential of edible insects to increase the intake of minerals, which are well reported to be deficient and causing severe public health problems in poor populations [45]. Given the worldwide deficiencies of these minerals among human population groups [46], insect-fortified cookies would supply the amount of iron and zinc required for basic body functions. Further, the bioavailability of minerals from insects is likely to be higher than that from plant foods because their nutrients are easily assimilated by the human body and there are no antinutritional factors such as phytic acid and oxalic acid in insects [47,48]. Previous studies found an appreciable concentration of minerals: calcium, iron, magnesium, copper, potassium, sodium, and zinc, and a particularly high iron content present in termites (*syntermes* soldiers) [49]. Chakravorty et al. [50] also reported that insects; *Oecophylla smaragdina* and *Odontotermes* sp. can serve as a source of micronutrients such as Fe, Zn, Cu, and Mn. Therefore, the consumption of insects should be encouraged, especially among rural communities with low animal protein intake, to contribute to meeting their nutritional requirements.

### 4.3. Amino Acid Profile

Lysine was significantly higher in cookies fortified with the sorghum–termite blend. The increase in lysine could also be attributed to the addition of the insect (termite) meal. It has been reported that the most concentrated essential amino acid found in termites was lysine [51]. As stated earlier, cereal grains are important staples in diets globally but are generally lysine deficient [39]. Therefore, supplementing cereal-based foods with insect is recommended as it would improve the lysine content of the foods. The lysine content reported in this study accounted for 80% of the recommended intake for children and 100% requirements for adults. The fact that insects are a traditional food in most developing regions, including SSA [52] is an advantage. Tryptophan and threonine known to be deficient in cereal proteins were also significantly higher in cookies fortified with the sorghum and termite meal. Tryptophan content was lowest in the control (10 mg/100 g) and ranged between 18 mg/100 g and 32 mg/100 g in cookies fortified with the sorghum–termite blend. Threonine content in cookies fortified with the sorghum–termite blend was between 30 mg/100 g and 46 mg/100 g, compared with 21 mg/100 g in the control. High concentrations of methionine and cysteine were also found (20 to 29 mg/100 g and 22 to 33 mg/100 g, respectively), in comparison to the control (18 mg/100 g and 18 mg/100 g methionine and cysteine, respectively). Histidine content ranged from 21 to 43 mg/100 g. Histidine is a precursor of histamine, which is present in small quantities in cells. Histamine communicates messages to the brain, triggers the release of stomach acid to aid digestion, and is released after an injury or allergic reaction as part of the body’s immune response [53]. Further, children grow poorly if there is an absence of histidine in their diet [53]. Therefore, cookies fortified with sorghum–termite blend developed in this study could be a good source of histidine required by children. Isoleucine (35 to 46 mg/100 g), leucine (48 to 63 mg/100 g), phenylalanine (33 to 44 mg/100 g), tyrosine (32 to 42 mg/100 g), and valine (42 to 47 mg/100 g) were also present in abundance. Overall, the concentrations of amino acids obtained in this study are higher compared with the concentrations found in meat sources such as beef, pork, and chicken meat [54]. Although it has been reported that the nutritional composition of insects may vary due to their feeding habits and harvesting season [51], values reported in this study could be largely dependent on the environmental factors from where the termites were purchased. As compared with the 2007 Food and Agriculture Organization (FAO)/World Health Organization (WHO) standard, the concentrations of the essential amino acids in all cookies fortified with the sorghum–termite blend were generally higher than the pattern of amino acid requirements for both children and adults [55] (Table 4). Hence, the developed cookies would be able to contribute to the essential amino acids in the human diet. Although most of the amino acids reported exceeded the requirements, they can be viewed as beneficial, especially for population groups whose staple diet consists of maize and wheat, which may lack some of the important amino acids [56].

### 4.4. In Vitro Protein Digestibility

In vitro protein digestibility (IVPD) ranged from 67% to 83% (Table 5). The reason for the higher IVPD in the cookies fortified with the sorghum–termite blend compared with the control is likely due to the higher digestibility of insect protein. Insect protein is highly digestible, and a range of 77% to 98% digestibility has been reported [39,57]. The results of this study are similar to the results reported by Ajayi [58], who reported high digestibility for winged termites (83.41%) and soldier termites (81.10%).

### 4.5. Physical Characteristics

The results show that as the sorghum–termite blend substitution level increased, cookie diameter decreased. Cookies containing 60% of the sorghum–termite blend had the lowest spread factor. The experimental cookies had a lower spread factor relative to the control most likely due to dilution of gluten, which is essential in the expansion of baked products [59,60,61].

### 4.6. Texture and Colour

The decrease in hardness and high fracturability of cookies fortified with the sorghum–termite blend may be attributed to dilution of gluten in the experimental cookies, because sorghum and termite do not contain gluten proteins. Gluten, which is formed during the dough mixing process and coagulated into a fiber-like foam, is responsible for the mechanical structure of baked products [61]. Furthermore, coarse particles may have been introduced by the increased fiber content of cookies, interfering with the homogeneity of the dough and cookie structure, thereby resulting in lower hardness values [62]. Additionally, the high crumbliness and fragility of the experimental cookies could also be due to high level of bran and the absence of gluten [63]. Table 7 shows that the 100% wheat cookies (control) were the hardest and least fracturable of all the cookies. The C0 (control) recorded the lowest fracturability value, while the maximum fracturability value was recorded for C60 (45% sorghum and 15% termite substitution level). Higher fracturability for cookies enriched with fiber has also been reported [64]. A previous study by Awobusuyi et al. [65], reported that fortifying wheat with a sorghum–insect meal did not compromise the product quality or acceptability, as the texture of the cookie samples containing the sorghum–termite meal was liked and rated the same as that of the control (100% wheat cookies).

The cookies darkened (decreasing Hunter L* values) with increased concentration of the sorghum–termite blend. This is likely due to the darker colour of the sorghum insect blend compared with wheat flour. Awobusuyi et al. [65], reported that the acceptability of sorghum–insect cookies and the colour acceptability of cookie samples supplemented with 5% sorghum–termite meal was higher when compared with the control (100% wheat biscuits) and cookies with higher concentrations of termites. It has also been reported that cookies made with whole meal sorghum resulted in cookies with a darker colour [63]. In addition, the increased protein content of the experimental cookies would result in production of higher levels of Maillard reaction products, the majority of which are brown pigments [66].

### 4.7. Water and Oil Absorption Properties of Cookie Flours

The results revealed that the higher the sorghum–termite blend substitution level, the higher the water absorption capacity (WAC). Water absorption capacity is a product’s ability to interact with water under restricted conditions [67]. Previous reports have suggested that flours with high water absorption capacity as the composite flours of this study would be beneficial in bakery products, as this could prevent staling by reducing moisture loss [68]. Similarly, oil absorption capacity (OAC) increased with increasing substitution level of sorghum–termite blend. Oil absorption capacity (OAC) refers to the capability of flour to absorb oil [69]. This is vital because oil acts as a flavour retainer and improves the mouth feel of cookies [70]. The observed trend of an increase in OAC with an increase in the concentration of the termite meal in biscuits may be attributed to the high protein content of the termite meal. The main chemical component affecting OAC in foods is protein, which is composed of both hydrophilic and hydrophobic parts. Hydrophobic proteins possess superior binding of lipids—non-polar amino acid side chains predominant in hydrophobic proteins can form a hydrophobic interaction with hydrocarbon chains of lipids, and thereby increase OAC [71,72]. The blends in this study are potentially valuable in the structural interaction in food, which is also important in developing new food products and the extension of shelf life, particularly in baked foods or other food products where fat absorption is desired [73,74].

### 4.8. Bulk Density of Cookie Flours

The results show that all flours containing the sorghum–termite blend had relatively higher packed bulk density (PBD) and loose bulk density (LBD) than the control. This indicated that the sorghum–termite blend had a higher bulk density than wheat flour. As explained earlier, bulk density provides information on the porosity of a product and can affect the choice and design of the packaging materials [42].

## 5. Conclusions

Cookies fortified with a sorghum–termite blend have the potential to serve as a protein, energy, and nutrient-rich supplementary food to address PEM. The results of the present study suggest that a sorghum–termite blend can be successfully incorporated into cookies up to a level of 60% (15% insect) and yield cookies of high nutritional value. The contribution of the experimental cookies to dietary iron, zinc, and lysine would be of particular significance as deficiencies of these nutrients remain a problem in Africa, especially in countries in SSA.

## Figures and Tables

**Table 1:** Ratios of ingredients (wheat:sorghum and termite) for cookie formulation.

Ingredient	Relative Concentration (% *w/w*)			
Wheat flour	100	80	60	40
Sorghum meal	0	15	30	45
Termite meal	0	5	10	15
Identity of cookie sample	C0 (control)	C20	C40	C60

Sorghum:termite meal (ratio 3:1) replaced wheat flour at 0, 20, 40, and 60% (*w*/*w*) levels.

**Table 2:** The sorghum–termite blend on the proximate composition (g/100 g) and gross energy (kJ) of cookies ^1^.

Sample	Ash	Protein	Fat	CHO	Dietary Fiber	Energy
C0	1.7 ^d^ ± 0.5	10.5 ^d^ ± 0.4	14.3 ^d^ ± 0.4	54.4 ^a^ ± 0.4	8.3 ^d^ ± 0.5	1180.2 ^d^ ± 0.5
C20	3.5 ^c^ ± 0.6	36.4 ^c^ ± 0.4	22.3 ^c^ ± 0.5	23.4 ^b^ ± 0.4	13.2 ^a^ ± 0.5	2032.8 ^c^ ± 0.5
C40	4.0 ^b^ ± 0.5	38.3 ^b^ ± 0.5	25.2 ^b^ ± 0.4	19.4 ^c^ ± 0.5	10.3 ^c^ ± 0.4	2108.4 ^b^ ± 0.3
C60	4.2 ^a^ ± 0.4	41.0 ^a^ ± 0.4	28.2 ^a^ ± 0.4	17.2 ^d^ ± 0.5	13.0 ^b^ ± 0.5	2217.6 ^a^ ± 0.2

Mean (±*SD*) of three determinations; CHO: glycemic carbohydrates; Means with different superscripts in a column vary significantly (*p* < 0.05); ^1^ Values are on dry matter basis. kJ; refers to Kilojoules; C0: control (100% wheat cookies); C20: 15% sorghum and 5% termite substitution level; C40: 30% sorghum and 10% termite substitution level; C60: 45% sorghum and 15% termite substitution level.

**Table 3:** Selected mineral elements in cookies fortified with sorghum–termite blend (mg/100 g) ^1.^

Minerals	C0	C20	C40	C60	Recommended Daily Intake ^2^	
					Children	Adults
Ca	2.5 ^d^ ± 0.4	5.5 ^c^ ± 0.5	8.6 ^b^ ± 0.5	10.8 ^a^ ± 0.5		
Fe	2.4 ^d^ ± 0.6	28.5 ^c^ ± 0.4	34.2 ^b^ ± 0.5	37.4 ^a^ ± 0.4	4.2	19.6
Mn	1.2 ^d^ ± 0.5	13.2 ^c^ ± 0.4	17.3 ^b^ ± 0.4	24.5 ^a^ ± 0.4		
Cu	0.8 ^d^ ± 0.6	1.4 ^c^ ± 0.5	2.4 ^b^ ± 0.5	3.8 ^a^ ± 0.6	0.9	1.3
K	1.8 ^d^ ± 0.6	12.5 ^c^ ± 0.4	18.6 ^b^ ± 0.5	22.9 ^a^ ± 0.4		
Na	0.8 ^d^ ± 0.6	8.1 ^c^ ± 0.5	13.5 ^b^ ± 0.6	16.9 ^a^ ± 0.5		
Zn	2.5 ^d^ ± 0.5	8.4 ^c^ ± 0.6	10.4 ^b^ ± 0.4	14.8 ^a^ ± 0.4	2.4	2.5
P	0.8 ^d^ ± 0.5	22.5 ^c^ ± 0.5	31.2 ^b^ ± 0.4	37.6 ^a^ ± 0.5		
Mg	1.8 ^d^ ± 0.4	24.3 ^c^ ± 0.5	29.6 ^b^ ± 0.6	33.5 ^a^ ± 0.5		

Mean (±*SD*) of three determinations. Means with different superscripts in a column vary significantly (*p* < 0.05). ^1^ Values are on dry matter basis. ^2^ Food and Agriculture Organization (FAO)/World Health Organization (WHO) 2006 Recommended daily intake for children and adults. C0: control (100% wheat cookies); C20: 15% sorghum and 5% termite substitution level; C40: 30% sorghum and 10% termite substitution level; C60: 45% sorghum and 15% termite substitution level.

**Table 4:** Essential amino acid composition of cookies fortified with sorghum–termite blend (mg/100 g) ^1^.

Amino Acids	C0	C20	C40	C60	FAO Reference Pattern ^2^	
					Children	Adults
Histidine	15	21	32	43		15
Lysine	10	22	37	49	75	45
Tyrosine	18	32	36	42		
Cysteine	18	22	29	33		6
Tryptophan	10	18	24	32	4.6	6
Methionine	18	20	25	29	34	16
Isoleucine	28	35	40	46	37	30
Phenylalanine	25	33	39	44	34	30
Threonine	21	30	34	46	44	23
Leucine	27	48	58	63	56	59
Valine	27	42	45	47	41	39

^1^ Values are on dry matter basis; ^2^ Food and Agriculture Organization/World Health Organization (2007). C0: control (100% wheat cookies); C20: 15% sorghum and 5% termite substitution level; C40: 30% sorghum and 10% termite substitution level; C60: 45% sorghum and 15% termite substitution level.

**Table 5:** In vitro protein digestibility of cookies fortified with sorghum–termite blend.

Samples	IVPD g/100 g	% Increase
C0	67 ^d^ ± 0.4	-
C20	73 ^c^ ± 0.2	8.95
C40	79 ^b^ ± 0.2	17.9
C60	83 ^a^ ± 0.3	23.8

Mean (± *SD*) of three determinations. Means with different superscripts in a column vary significantly (*p* < 0.05). C0: control (100% wheat cookies); C20: 15% sorghum and 5% termite substitution level; C40: 30% sorghum and 10% termite substitution level; C60: 45% sorghum and 15% termite substitution level.

**Table 6:** Physical qualities of cookies fortified with sorghum–termite blend.

Samples	Weight (g)	Diameter (mm)	Thickness (mm)	Spread Factor
C0	29.5 ^a^ ± 0.5	45.8 ^a^ ± 0.2	7.3 ^c^ ± 0.6	6.2 ^a^ ± 0.4
C20	27.4 ^b^ ± 0.7	44.4 ^b^ ± 0.4	7.5 ^c^ ± 0.7	5.9 ^b^ ± 0.7
C40	25.4 ^c^ ± 0.7	42.3 ^c^ ± 0.6	7.7 ^b^ ± 0.4	5.5 ^c^ ± 0.5
C60	24.5 ^c^ ± 0.7	41.6 ^c^ ± 0.5	7.9 ^a^ ± 0.5	5.2 ^c^ ± 0.2

Mean (± *SD*) of three determinations. Means with different superscripts in a column vary significantly (*p* < 0.05). C0: control (100% wheat cookies); C20: 15% sorghum and 5% termite substitution level; C40: 30% sorghum and 10% termite substitution level; C60: 45% sorghum and 15% termite substitution level.

**Table 7:** Texture and colour of cookies fortified with sorghum–termite blend.

Sample	Hardness (N)	Fracturability (mm)	Colour		
			Hunter L*	a*	b*
C0	36.4 ^a^ ± 5.5	0.31 ^d^ ± 0.8	47.3 ^a^ ± 0.5	12.7 ^d^ ± 0.6	8.1 ^d^ ± 0.6
C20	29.4 ^d^ ± 8.6	0.64 ^c^ ± 1.2	46.2 ^b^ ± 1.2	14.5 ^c^ ± 0.8	8.3 ^c^ ± 0.7
C40	31.7 ^c^ ± 9.9	0.72 ^b^ ± 0.3	44.5 ^c^ ± 0.5	17.4 ^b^ ± 1.2	8.4 ^b^ ± 1.5
C60	32.1 ^b^ ± 0.4	1.14 ^a^ ± 0.1	41.2 ^d^ ± 0.6	18.8 ^a^ ± 0.7	8.6 ^a^ ± 0.7

Mean (± *SD*) of three determinations. Means with different superscripts in a column vary significantly (*p* < 0.05). L* = Lightness, a* = Redness, b* = Yellowness. C0: control (100% wheat cookies); C20: 15% sorghum and 5% termite substitution level; C40: 30% sorghum and 10% termite substitution level; C60: 45% sorghum and 15% termite substitution level.
